# Phase Separation, Reaction Equilibrium, and Self-Assembly in Binary Telechelic Homopolymer Blends

**DOI:** 10.1021/acs.macromol.3c01653

**Published:** 2023-12-13

**Authors:** Daniel L. Vigil, Amy Zhang, Kris T. Delaney, Glenn H. Fredrickson

**Affiliations:** † Department of Chemical Engineering, University of California, Santa Barbara, California 93106, United States; ‡ Materials Research Laboratory, University of California, Santa Barbara, California 93106, United States

## Abstract

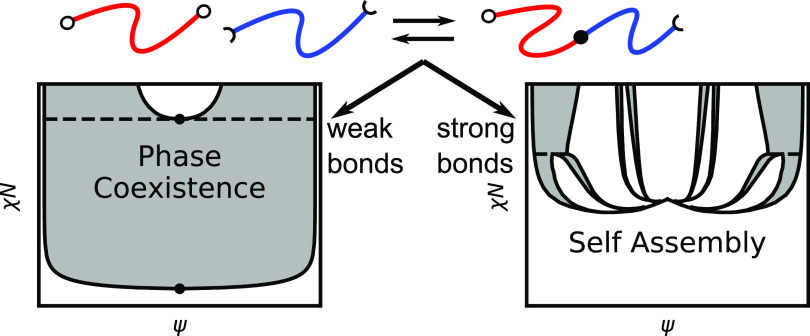

We study a binary blend of telechelic homopolymers that can form reversible AB-type bonds at the chain ends. Reversibly bonding polymers display novel material properties, including thermal tunability and self-healing, that are not found in conventional covalently bonded polymers. Previous studies of reversibly bonding polymer systems have been limited by the computational demand of accounting for an infinite number of possible reaction products in a spatially inhomogeneous, self-assembled structure. We demonstrate that newly developed theoretical models and numerical methods enable the simultaneous computation of phase equilibrium, reaction equilibrium, and self-assembly via self-consistent field theory. Phase diagrams are computed at a variety of physically relevant conditions and are compared with nonreactive analogues as well as previous experimental studies of telechelic polymer blends.

## Introduction

Block copolymers (BCPs) are a major industrial platform due to their highly tunable properties via self-assembly and the ability to compatibilize dissimilar polymers. A major triumph of polymer physics is the ability to predict when melts and solutions of homopolymers and copolymers will mix into a single liquid, macroscopically phase separate into two liquids,^1,2^ or self-assemble into a solid-like microstructure.^3^ One important tool for predicting this phase behavior is numerical self-consistent field theory (SCFT), which is particularly effective at computing the free energies of the self-assembled microstructures.^4^ Numerical advances over the last 30 years have enabled efficient simulation of complex self-assembled structures, including Frank–Kasper sphere phases.^5,6^ The standard approach for numerical SCFT uses an auxiliary field (AF)-based model, which decouples chain interactions via a set of local potential fields.^4^ This approach is well suited to polymer systems with a predefined distribution of components in the mixture or “quenched” systems.^7^ In recent years, however, there has been significant interest in supramolecular interactions, where polymers and small molecules can form and dissociate bonds reversibly and are in a dynamic equilibrium. These types of interactions appear in both synthetic and biological polymers, including intrinsically disordered proteins^8^ and polymers functionalized with acid and base groups,^9,10^ multiple hydrogen bonds,^11^ and ligands that bind to metals.^12^ Supramolecular interactions are also of industrial interest as some commodity polymers, such as thermoplastic polyurethanes, can reversibly dissociate and form bonds at elevated temperatures.^13−15^ Supramolecular interactions can also lead to exotic phase behavior, including re-entrant phase transitions,^16^ and can also be leveraged to make thermally tunable^10^ and self-healing materials.^17^

Some authors have studied supramolecular polymer systems with theories similar to those of AF-SCFT by coupling the theories to analytical approximations. In particular, the phase separation and gelation of blends of linear polymers with associating groups along the length of the polymer have been studied by multiple groups.^18−21^ These analytical approaches have not been used to examine the microstructures formed during self-assembly, however, even though theory and experiments^9,16,22−24^ show that self-assembly occurs in supramolecular polymer blends. One group of authors used molecular dynamics simulations to examine microphases and were able to study domain sizes, but they did not create comprehensive phase diagrams.^19^

There have been attempts to extend the numerical AF-SCFT approach to supramolecular polymers.^25−29^ For systems in which only a finite set of products can be formed, the approach works well, and phase diagrams that include microphases have been computed. One example is a supra-diblock system in which two dissimilar homopolymers each have one functional group on a chain end that can link together to form a diblock.^26^ In many systems, however, there are an unlimited number of possible products. This includes telechelic and network-forming polymers. For these systems with an unlimited set of products, the AF-SCFT approach relies on generating functionals to enumerate the linear and tree-like products, but neglects ring and loop products.^28^ Even with these approximations, the approach is limited by the computational expense of numerically solving integral equations inside the SCFT field iteration and has not been widely deployed. In particular, comprehensive phase diagrams have not been developed for any system with an unlimited set of reaction products.^29^

Recent theoretical developments have produced an alternative to the AF approach that instead represents polymers via coherent state (CS) fields.^30,31^ These CS models are particularly effective for supramolecular systems as they can represent all possible reaction products with the proper weighting, even when there are an unlimited number of products.^32^ The models are of finite order in the CS fields and do not rely on any approximate scheme for partially summing product contributions. Recent numerical advances have enabled efficient simulation of these models as well,^33^ though these algorithms have not yet been applied to supramolecular polymers.

In this work, we combine these theoretical and numerical advances to demonstrate that CS-SCFT can be used to construct full phase diagrams incorporating reaction equilibrium for supramolecular polymers. As a model system, we consider a binary blend of telechelic homopolymers that can form AB-type bonds. This system can form arbitrarily long alternating AB-type BCPs, which makes it intractable to AF-SCFT calculations. The full CS theory also accounts for ring polymers that can be formed, but the mean field approximation invoked for SCFT does not enumerate these products.^34^ We compute full phase diagrams that include phase coexistence between disordered phases and microphases, including the body-centered cubic (BCC) sphere phase and the double gyroid network phase (GYR). These phases require 3D calculations, which have not been performed for supramolecular BCPs with infinite product sets before.

With our approach, we are able to demonstrate three different regimes of phase behavior depending on the relative strength of the bond equilibrium and the phase segregation strength. When bonds are weak, the system behaves similarly to a nonreactive homopolymer blend. In the opposite limit, when bonds are strong, the system behaves like a pure BCP melt or BCP-homopolymer blend, depending on the stoichiometry of the system. In the intermediate regime, we observe a complex interplay between macroscopic phase separation and microphase segregation characteristic of the region around a Lifshitz point. In addition to phase diagrams, we are able to predict the reaction equilibrium in the system as well as the microphase structure, including domain spacing. These results demonstrate the range of possible phase behavior and will help guide experimental polymer chemists who use supramolecular chemistry in polymer blends.

## Model and Methods

We consider a binary melt blend of A and B telechelic homopolymers in which each A-type chain end can reversibly bind to a single chain end of type B (heterobonding motif). A CS model for such a system in the canonical ensemble is
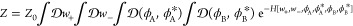
1
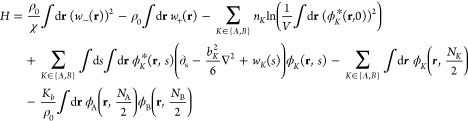
2Here, *Z* is the partition function, *Z*_0_ is the ideal gas partition function, and *H* is the effective Hamiltonian. Normalizing denominators for the functional integrals have been absorbed into *Z*_0_.^31^ The Hamiltonian depends on four CS fields (φ_A_, φ_A_*, φ_B_, and φ_B_*) and two AFs (*w*_+_ and *w*_–_), so the model may be referred to as a hybrid AF-CS model. The AFs are defined over all of space **r**, whereas the CS fields depend on **r** as well as the chain contour position, *s* ∈ [0, *N*_K_/2]. Each term in the Hamiltonian has a simple physical interpretation. The logarithmic terms on the second line create the appropriate number of homopolymer precursor chains, *n*_A_ or *n*_B_, depending on species. The terms contain factors of , which creates two polymer arms at a core *s* = 0 contour position. The third line of the Hamiltonian is responsible for propagating these arms outward from the core in *s* using the appropriate chain statistics. In this work, we consider flexible continuous Gaussian chains, which leads to the diffusive-type operator ∂_s_ – ∇^2^ that appears in the Hamiltonian. The statistical segment length can be set for each species via *b*_K_, but we consider only *b*_A_ = *b*_B_ = 1 in this work. While an arm is being propagated, it experiences the relevant species potential field *w*_A_ or *w*_B_, which are related to the *w*_±_ AFs via a simple linear transformation
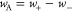
3
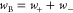
4

An important feature of the model is that the integration path of *w*_–_ is over real values, while *w*_+_ is pure imaginary. The species fields *w*_A_ and *w*_B_ are thus complex-valued, as is the Hamiltonian. After *N*_K_/2 segments, the arm is terminated via the first term in the last line. Because the polymer was initialized as a star with two arms, this creates a linear chain with a total length of *N*_K_. Unless otherwise specified, we choose the following equation: *N*_A_ = *N*_B_. The final term in the Hamiltonian enables an A and a B polymer to link together at their chain ends and has an associated equilibrium constant *K*_b_. In addition to creating the diblock, this term also creates all higher-order products, including triblock, tetrablock, and so on. Finally, the first two terms in the model represent the nonbonded interactions between polymer segments in the model. The first introduces a Flory–Huggins interaction between A and B segments parametrized by χ and the second enforces incompressibility at a segment number density ρ_0_ = (*n*_A_*N*_A_ + *n*_B_*N*_B_)/*V*, where *V* is the total volume of the system and *n*_K_ is the number of species *K* telechelic polymers.

It is possible to remove the AFs by explicit evaluation of the *w*_±_ integrals, resulting in a “pure” CS model with additional interaction terms that are fourth order in the CS fields.^30,32^ However, previous work has found the hybrid representation more amenable to numerical simulation so we retain this form of the model for the present study.^33^ Although we have presented the model here based on physical arguments, it is possible to derive it rigorously from an AF model, as is demonstrated in the literature.^30,31^ Correspondingly, it is possible to show that every copolymer product, both linear and cyclic, is accounted for correctly by performing a perturbation expansion in the powers of *K*_b_.

It is worth discussing the relationship between the model parameters and the parameters under experimental control. In the model, polymer segments are defined to have equal volume, so the volume fraction of a given segment type (A or B) is ψ_K_ = *n*_K_*N*_K_/(*n*_A_*N*_A_ + *n*_B_*N*_B_). Two model parameters have important temperature dependences: χ and *K*_b_. For most polymer pairs, the chi parameter can typically be fitted to an expression of the form
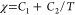
5where *T* is temperature and *C*_1_ and *C*_2_ are constants that may depend on the composition ψ_A_ of the blend.

The equilibrium constant follows the relation
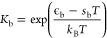
6where *k*_B_ is Boltzmann’s constant, ϵ_b_ is the enthalpy of reaction, and *s*_b_ is the entropy of reaction. We assume for simplicity that both ϵ_b_ and *s*_b_ are independent of temperature.

The model presented above can be applied to any experimental system for which there is a known temperature dependence for χ and *K*_b_. In this work, rather than specializing on specific chemistries, we make an approximation to examine general trends. We assume that χ is inversely proportional to temperature (*C*_1_ = 0 in eq 5) and that the entropy of reaction *s*_b_ = 0. With these approximations, χ and *h* = ln(*K*_b_) are both inversely proportional to temperature and their ratio is independent of temperature. This allows us to use χ as an inverse temperature scale and *h*/χ as a chemistry-dependent property that represents the strength of bonding compared to phase separation.^26,27^

As a final note, it is conventional in unreactive BCPs to specify χ*N* rather than χ as it is the combined grouping that controls the phase behavior for linear chains. This is no longer true in the telechelic model considered here, as the reaction can only occur at end groups. Changing *N*_A_ or *N*_B_ changes the concentration of these end groups, breaking the universal phase behavior for a fixed χ*N*. Nevertheless, to match convention, we will use the combined grouping χ*N*_A_ and *h*/χ*N*_A_ and fix *N*_A_ = 100. Although this reduces the generality of the results presented here, it is well understood how changing *N* affects phase behavior and reaction equilibrium based on previous literature results.^26^

We now turn to physical observables that can be computed by field operators. The first of these is the segment density of the A or B species
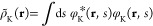
7a similar quantity that only considers the unreacted end segments of a chain can also be defined as an end density
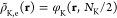
8

Finally, an operator for the local density of bonds is defined via
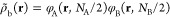
9

These operators give important information about the spatial distribution of the segments in the system. We are also concerned with bulk properties as well, including the total number of bonds
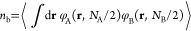
10and number of unreacted ends of a given species in the system
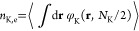
11

Here, the angle brackets denote an average over field configurations. Another useful quantity is the conversion of end groups, which is defined as
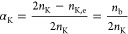
12

The conversion of end groups for species A and B is related because only AB-type bonds can form
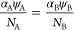
13

The internal stress of a blend can also be computed via a field operator. Such an operator is familiar in AF representations and has a similar form in the CS model. The full derivation of such an operator can be found in the Supporting Information of a recent publication by Fredrickson and Delaney,^35^ so we present the final result
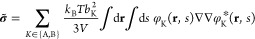
14

A final important operator is the chemical potential, which is required to construct phase-coexistence regions. For a given species, the excess chemical potential in units of the thermal energy *k*_B_*T* is
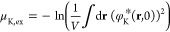
15

This chemical potential is in excess of the ideal gas chemical potential of a non-interacting reference system with no supramolecular bonds.

The previously discussed operators give average information about the reaction equilibrium, such as the conversion, but we would also like to know the distribution of products in the system, including how much of each type of BCP is formed. Unfortunately, there is no known operator using the CS fields that can be used to compute the number of each type of reaction product. It is possible, however, to compute the number of chains of a given species in an AF model formulated in the grand canonical ensemble. Although the unlimited number of reaction products makes it intractable to use the AF model to establish reaction equilibrium, we can use the CS model to obtain equilibrium field configurations and then evaluate operators in the AF model to quantify the number of any individual reaction product. The effective Hamiltonian for an AF model of the telechelic blend presented here is
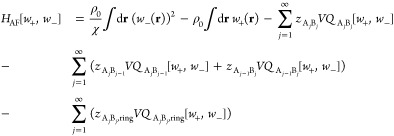
16and the number of each product can be computed via
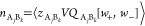
17

The first two terms of the effective Hamiltonian are equivalent to those of the CS model and represent Flory interactions and incompressibility. The remaining terms contain activities *z* and single-chain partition functions *Q*[*w*_+_, *w*_–_] for all the possible products that can be formed. The activities and single-chain partition functions can also be used to compute the number of each product. The products can be classified into three types: linear chains that are composed of an equal number of A and B chains and are terminated by one A chain and one B chain, linear chains with one excess A or B chain that are doubly A or B terminated, and ring polymers, which must have an equal number of A and B chains. One can demonstrate that the activity of each type of chain can be related to the activity of the A and B homopolymers, *z*_A_ and *z*_B_, and the equilibrium constant *K*_b_ via
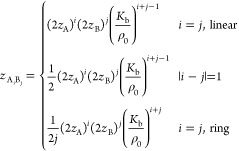
18the cases of this equation represent the different types of products, as discussed previously. The first two cases, which represent different types of linear chains, are nearly identical, only differing by a factor of 2. The products that are doubly A terminated or doubly B terminated are head–tail symmetric so their activity carries a factor of 1/2 to account for this degeneracy. Similarly, for ring polymers, there is a factor of 1/2*j* that accounts for the rotational symmetry of the molecule. There is also an additional factor of *K*_b_ that accounts for the extra supramolecular bond in a ring compared with a linear chain.

Although we have expressions for the activities of each product, we still require the activities of the homopolymers and also the values of the single-chain partition functions to compute the number of each product. The homopolymer activities can be computed from the chemical potential determined from canonical ensemble simulations with the CS model since *z*_A_ = ρ_0_ exp(μ_A_). All that remains then is to compute the ensemble average single-chain partition function value for each chain using equilibrium field configurations from the CS simulation. This would normally be prohibitively expensive since there are an infinite number of reaction products. We circumvent this issue by considering only BCPs containing up to 14 telechelic homopolymers. We note that the equilibrium field configurations obtained from the CS simulation account for all products, not just those considered in the truncated set. One can evaluate how much of the mass is accounted for with the truncated set of products by comparing the total number of polymers from the original CS canonical simulation and comparing it to the number of products computed from the AF approach. In all cases in this work, the truncation error is less than half a percent, unless otherwise noted. The truncated set can also be extended to larger products if it is found to be missing a significant fraction of the polymer mass.

The distribution calculation in this work is further accelerated by the fact that we limit ourselves to SCFT calculations and must evaluate each *Q* only at the final mean-field (saddle point) configuration obtained from the CS model. We discuss details of the numerical SCFT method in the following section. For the disordered phase, numerical simulations are not required and the single-chain partition function can be computed analytically under SCFT. Additionally, in the disordered phase, the activity of the A and B homopolymers can be related to the system composition analytically. We can then analytically compute the distribution of products in the disordered phase, which yields
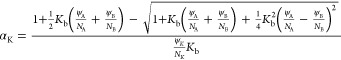
19
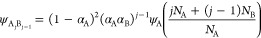
20
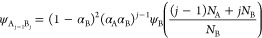
21
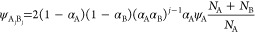
22Equation 19 gives the conversion of species A or B in the disordered phase, while eqs 20–22 provide expressions for the volume fractions of the different types of products that can be formed. The three types of products include A-terminated chains, B-terminated chains, and chains terminated with one A and one B telechelic. Note that SCFT does not account for rings. One can show analytically via an infinite summation that the volume fractions sum to unity, indicating that we have properly accounted for all products. The compositions are also consistent with a probabilistic interpretation of the product distribution. In the disordered state, the system is assumed to be well mixed, so the probability that an A chain end is reacted is α_A_, and the probability that it is unreacted is (1 – α_A_). The probability that an A telechelic has two unreacted ends is then , which is proportional to the amount of unreacted homopolymer in the blend, consistent with eq 20. To form an AB diblock requires an A telechelic with one unreacted end and one reacted end, generating the weight 2α_A_(1 – α_A_), where the factor of 2 accounts for the indistinguishability of the two ends. The reacted end must be linked to a B block, which then has its other end unreacted and is associated with a factor of (1 – α_B_). The volume fraction of an AB diblock should then be proportional to 2α_A_(1 – α_A_)(1 – α_B_), which is consistent with eq 22. One can extend this logic to higher-order products; for example, an ABA triblock has the expected factor of  and is properly accounted for in eq 20.

### Numerical Self-Consistent Field Theory

Numerical SCFT is an approximation to the full model in which we only consider a single saddle point configuration that satisfies the equations
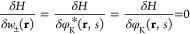
23

The free energy of the system is equal to the value of the effective Hamiltonian evaluated in these saddle-point field configurations.

To obtain the saddle-point fields, we use a previously developed algorithm.^33^ The simulations are conducted in orthorhombic unit cells with periodic boundary conditions. The phases considered in this work are disordered liquid (DIS), LAM, hexagonally packed cylinders (HEX), BCC spheres (BCC), and a GYR. It is possible that other phases, such as close-packed spheres or Frank–Kasper sphere packings may occur, but we do not consider them here. The 3D phases, BCC and GYR, were spatially discretized using a 64^3^ mesh, while the 2D HEX phase was discretized with a 64 by 108 grid and the 1D LAM phase used 128 grid points. In all calculations, the polymer contour was discretized with 11 sample points on a Chebyshev grid across the interval [0, *N*_K_/2] for each polymer. The SCFT equations were nondimensionalized using  as a reference length scale and *k*_B_*T* as an energy scale. All calculations were run until the *L*_2_ norm of the first variation of the dimensionless Hamiltonian was less than 1 × 10^–7^ with respect to all fields. A variable cell shape algorithm was employed to obtain stress-free configurations,^36^ with stress quantified using a nondimensionalized version of the CS stress operator presented earlier. All calculations were run until the stress was less than 10^–6^.

### Gibbs Ensemble

For the blend system considered here, it is possible for the system to macroscopically separate into multiple coexisting phases. Although there are many possible products in the melt, all are formed from the two starting macromonomers via the reaction equilibrium. Thus, the only independent chemical potentials are those of the two telechelic homopolymers, and only two-phase coexistence regions are possible. This is in contrast to unreactive A homopolymer, B homopolymer, and diblock blends, which display three-phase coexistence.^37−40^ To determine binodals of phase coexistence, we use the Gibbs ensemble approach pioneered by Panagiotopoulos in the context of particle simulations.^41,42^ The Gibbs ensemble approach was later adapted to field theoretic simulations by Riggleman and co-workers^43^ and eventually specialized to SCFT of incompressible blends by Mester and coauthors.^44,45^ We employ the Gibbs ensemble method of Mester et al. in this work. In a Gibbs ensemble, the system is divided into two subsystems, with each containing a different phase. The temperature, number of polymers, and total volume of the system are fixed. We adjust the composition and volume of each subsystem to equalize the osmotic pressure and chemical potential between the two subsystems, subject to the mass and volume conservation constraints of the total system. This approach only requires one calculation per temperature along each binodal, in contrast to other approaches such as common tangent or grand canonical ensemble that require many calculations.^46^

## Results and Discussion

### Weak Bonding

**Figure 1:**
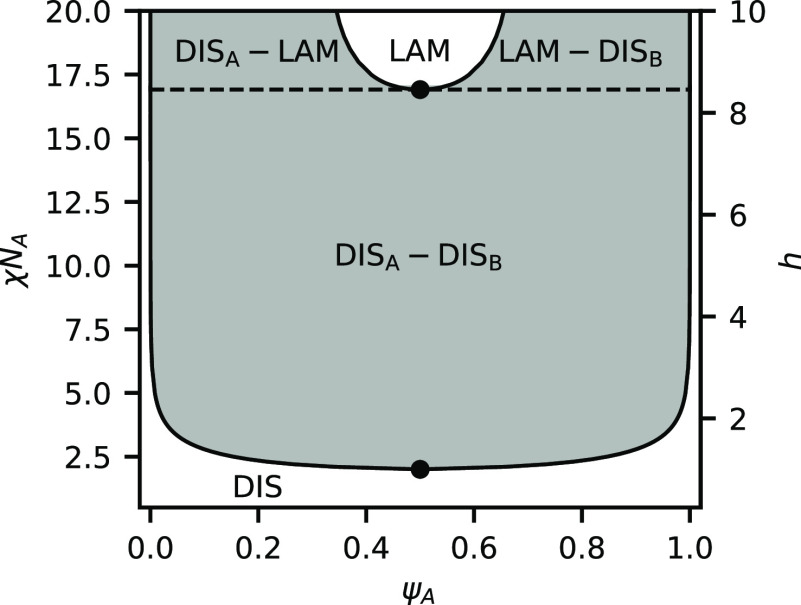
Phase diagram for a binary blend of heterobonding telechelic homopolymers at *h*/χ*N*_A_ = 0.5. Shaded regions indicated two-phase coexistence, while white areas indicate a single-phase. Critical points are indicated with solid dots.

We first examine a case where bonding is weak compared to the tendency for phase separation and set *h*/χ*N*_A_ = 0.5. Figure 1 shows the phase diagram in the space of χ*N*_A_, which is inversely proportional to temperature, and the volume fraction of A segments ψ_A_. The phase diagram is dominated by a region of phase coexistence between an A-rich DIS and a B-rich DIS. This is very reminiscent of an unreactive homopolymer binary blend, and the critical point in the reactive system is quite close to that of the unreactive system (χ*N*_A_ = 2.0, ψ_A_ = 0.5). At sufficiently high χ*N*_A_, a region emerges where the lamellar phase is stable, which is flanked by regions of coexistence with disordered phases. Because the ratio of *h*/χ*N*_A_ is fixed, increasing χ*N*_A_ also increases the equilibrium constant, favoring BCP product formation.

**Figure 2:**
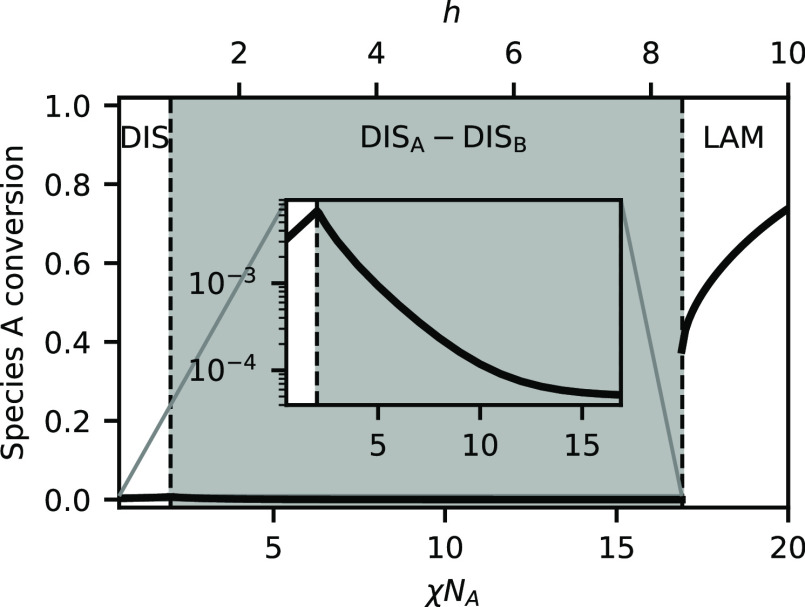
Species A conversion, α_A_, versus χ*N*_A_ (solid dark line), at *h*/χ*N*_A_ = 0.5 and ψ_A_ = 0.5. Shading and text labels indicate the stable phase(s). Vertical dashed lines indicate phase boundaries. The inset shows an expanded view of the conversion for χ*N*_A_ < 16.9 on a logarithmic *y*-axis.

To better understand the phase behavior, it is useful to examine the reaction equilibrium in the blend. Figure 2 shows the conversion of species A as χ*N*_A_ is varied at a fixed total composition of ψ_A_ = 0.5. Because the system is symmetric, at this composition α_A_ = α_B_ and we plot only plot α_A_. The inset of Figure 2 shows that below the critical point near χ*N*_A_ = 2, the conversion is quite low , but increases with increasing χ*N*_A_. This trend reverses at the critical point, however, and increasing χ*N*_A_ decreases the conversion in the system until χ*N*_A_ = 16.9. We attribute this to the increasing strength of phase segregation in this region. The conversion decreases because, as χ*N*_A_ increases, there are fewer and fewer B chains present in the A-rich phase with which A chains can react, and vice versa. Finally, at χ*N*_A_ = 16.9, the lamellar phase forms, and there is a sudden increase in the conversion. The equilibrium constant *K*_b_ has increased sufficiently that it is now energetically favorable to remix the two liquids so that they can form BCPs, which then self-assemble into a lamellar structure. There is still a significant amount of homopolymer in the mixture, but it can segregate to the interior of the A and B domains, while the BCPs act like surfactants at the interface.

Just past the critical point, the domain spacing of the lamellar phase is quite large (≈8 R_g_) compared to a lamellar domain formed from pure AB diblocks of length 2*N*_A_ (5.5 R_g_) at the same χ*N*_A_. For a volume fraction of A less than 0.35 or greater than 0.65, the system exhibits phase coexistence between the lamellar phase and a disordered phase. The disordered phase is composed almost entirely of the majority component of the system and contains almost purely homopolymers and almost no BCPs. Rather than swelling the lamellar domain with all the excess homopolymer that exists because of stoichiometry, it is instead favorable to eject it into a separate phase and maintain a less swollen lamellar phase. Because our calculations invoke a mean field approximation, there are no thermal fluctuations and Helfrich repulsions that would promote unbinding for highly swollen LAM, possibly leading to a microemulsion.^47,48^

Further increasing χ*N*_A_ above 17 leads to further increased conversion and depletion of the remaining homopolymer in the system. This causes the domain size to decrease as there is less homopolymer to swell the system. We truncate this phase diagram at χ*N*_A_ = 20, but it is possible that other phases can form at even higher values of χ*N*_A_. The conversion provides a simple scalar description of the reaction equilibrium but does not provide information on what types of BCPs are present in the system. In Figure 4, we plot the distribution of products at ψ_A_ = 0.5 and χ*N*_A_ = 17 and χ*N*_A_ = 20.

**Figure 3:**
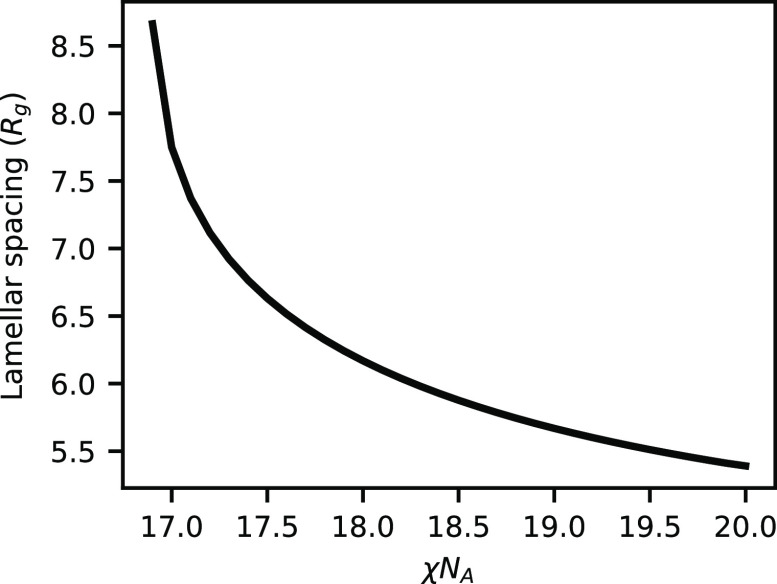
Domain spacing of the lamellar phase as a function of χ*N*_A_ at *h*/χ*N*_A_ = 0.5 and ψ_A_ = 0.5.

**Figure 4:**
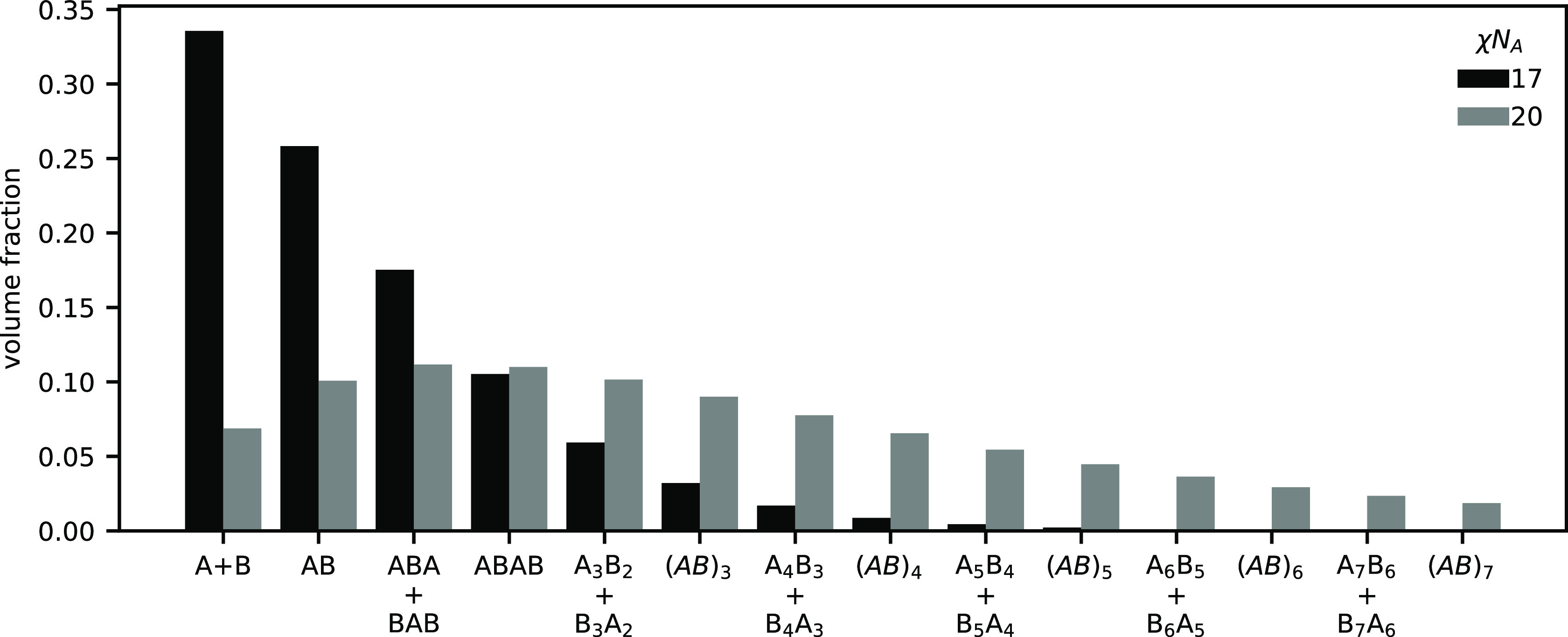
Distribution of products in the lamellar phase at *h*/χ*N*_A_ = 0.5, ψ_A_ = 0.5, and χ*N*_A_ = 17 or χ*N*_A_ = 20. Blend is self-assembled into LAM at both χ*N*_A_ = 17 and 20.

At χ*N*_A_ = 17, the distribution is dominated by the AB diblock and the individual homopolymers, with triblocks and the tetrablock also making meaningful contributions to the total volume of the system. Higher order BCPs quickly become irrelevant with increasing size; however, at χ*N*_A_ = 20, the distribution is significantly broadened and the amount of homopolymer is reduced by 80% compared to χ*N*_A_ = 17. The average product is also shifted to longer BCPs. Note that the products considered in Figure 4 account for only 93% of the mass, the remainder of which is composed of longer BCPs not included in the population analysis. The dramatic increase in average polymer size upon changing χ*N*_A_ from 17 to 20 may lead to significantly slowed dynamics in experimental systems and prevent the observation of equilibrium phases due to kinetic trapping.

Experimentally, there will also be ring polymers formed in the blend, although these are neglected by the mean field approximation of SCFT, as mentioned earlier. It is known that a melt of ring diblock copolymers has a larger value of χ*N*_ODT_ than a melt of linear diblock copolymers.^49^ Molecular dynamics simulations of blends of type A ring homopolymers and type B linear homopolymers show that the rings and linears undergo phase separation at larger values of χ*N* compared to blends of linear homopolymers.^50^ Together, these effects imply that including ring polymers in the blends produced by telechelic heterobonding homopolymers will likely stabilize the disordered phase and shift the ordered phase (LAM) window to larger values of χ*N*_A_.

### Strong Bonding

**Figure 5:**
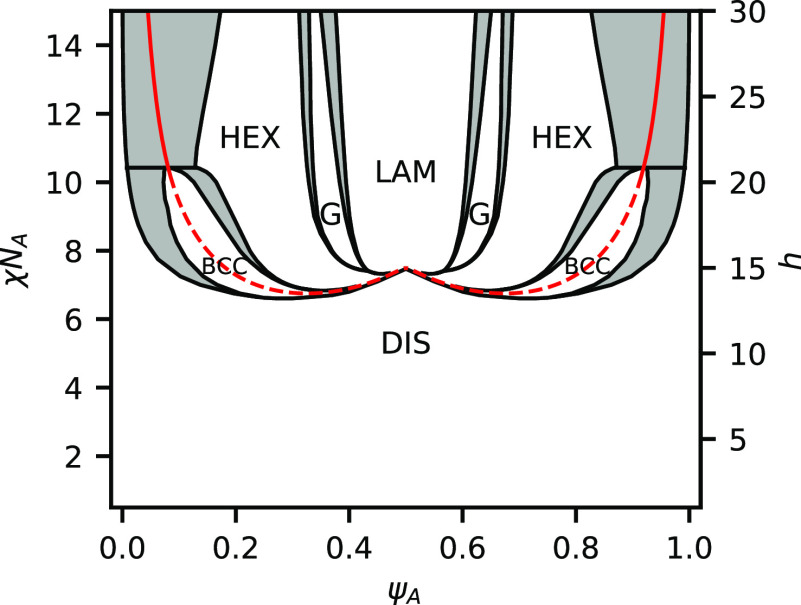
Phase diagram for a binary blend of heterobonding telechelic homopolymers at *h*/χ*N*_A_ = 2.0. Shaded regions indicate two-phase coexistence, while white regions indicate a single-phase is present. The present phases are a homogeneous DIS, LAM, GYR, HEX, and BCC packing of spheres. Red line indicates the RPA spinodal boundary. Dashed segments indicate instability at the nonzero wave vector. Solid segments indicate instability at wave vector *k* = 0.

We now consider a case where the equilibrium constant is large compared to the segregation strength and set *h*/χ*N*_A_ = 2, the phase boundaries for which are plotted in Figure 5. Contrary to the weak-bonding case, there is no large coexistence region between disordered phases. Instead, the phase diagram is dominated by regions of microphases separated by channels of phase coexistence. At the edges of the phase diagram, there are significant regions of phase coexistence between the microphase and the disordered phase. Similar to the weak-bonding case, it is favorable to eject excess homopolymer to avoid significantly swelling the domains. It is also possible that other sphere phases, such as close-packed spheres or Frank–Kasper phases, could be present in these regions, but we do not consider them in this work. While the relative position of the stable region for each microphase is similar to that of unreactive BCPs, there are some notable differences, including the fact that the BCC phase becomes unstable above χ*N*_A_ ≈ 10.5 and is replaced with a two-phase window between DIS and HEX. The shape of the order–disorder transition (ODT) is also quite different from unreactive BCPs and has a cusp in the center of the phase diagram, indicating that at ψ_A_ = 0.5, the mixture has a higher segregation strength χ*N*_ODT_ at the ODT than at ψ_A_ = 0.33 or ψ_A_ = 0.66.

Figure 5 also shows the spinodal boundary computed via the random phase approximation (RPA)^3,32^ in red. The dashed segments of the red line indicate nonzero wave vector *k* instability, which is characteristic of microphase separation. The solid section of the red line indicates a wave vector *k* = 0 instability, indicative of phase coexistence and possible macroscopic phase separation. The RPA is in good agreement with the full phase diagram from SCFT. The dashed segments of the RPA boundary, which indicate microphase separation, lie within the single-phase regions of the phase diagram. RPA also predicts a change from *k* ≠ 0 to *k* = 0 instability near the BCC-HEX-DIS triple point at χ*N*_A_ = 10.5. The solid section of the RPA line, which indicates macrophase separation, lies inside the DIS-HEX coexistence region. We now examine the reaction equilibrium and then explain the shape of the ODT.

**Figure 6:**
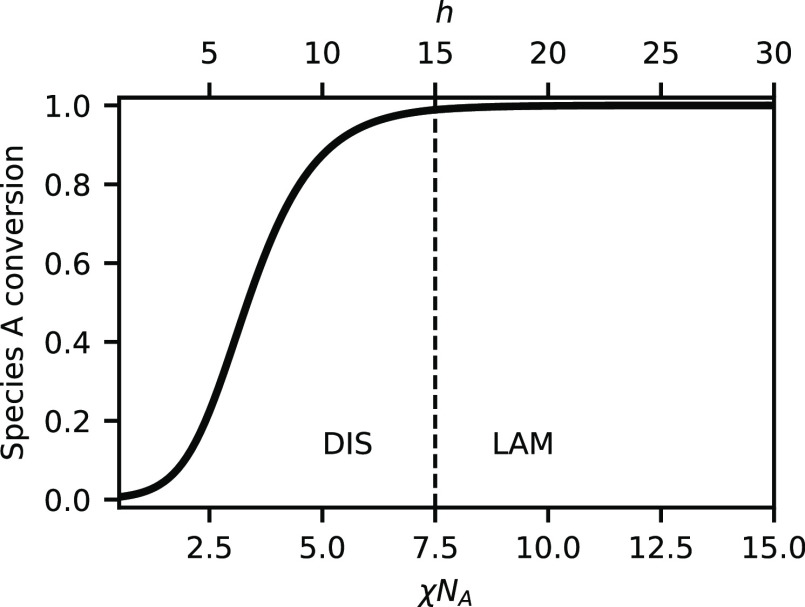
Species A conversion, α_A_, versus χ*N*_A_ at *h*/χ*N*_A_ = 2.0 and ψ_A_ = 0.5. Text labels the stable phase and the vertical dashed line indicates the phase boundary.

Figure 6 shows the conversion of species A with varying χ*N*_A_ at ψ_A_ = 0.5, analogous to Figure 2 for weak bonding. For strong bonding, the conversion of species A increases quite quickly with increasing χ*N*_A_ so that at χ*N*_A_ = 2 (the unreactive homopolymer blend critical point), α_A_ = 0.1. At this level of conversion, there is enough BCP present to compatibilize the A and B homopolymers and maintain a single disordered phase despite the relatively strong χ*N*_A_. Further increasing χ*N*_A_ continues to increase the conversion until it reaches near completion. Near the ODT at χ*N*_A_ = 7.45, the conversion in the disordered phase reached nearly 100%. Crossing into the lamellar phase continues this trajectory and the conversion stays near unity. One can repeat this exercise at other values of ψ_A_ to find that the minority species is nearly 100% converted at the ODT for all compositions.

**Figure 7:**
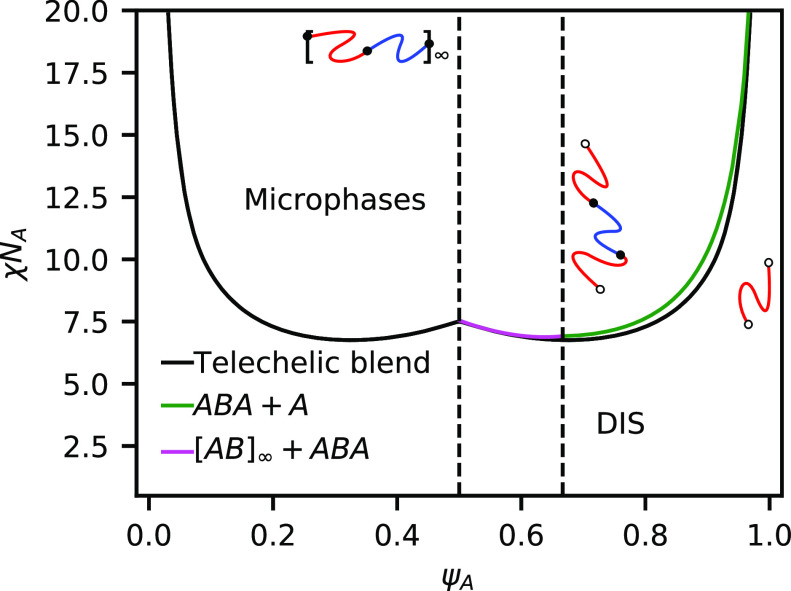
Order–disorder spinodals computed via RPA for the telechelic blend system at *h*/χ*N*_A_ = 2 (black), an unreactive binary blend of ABA triblock and A homopolymer (green), and an unreactive blend of ABA triblock with infinitely repeating (AB) multiblock (magenta).

Based on this information, we might approximate the system as being composed of all BCPs or copolymers plus a single excess homopolymer component, depending on stoichiometry. Using this assumption, we can rationalize the shape of the ODT based on the stoichiometry of the system. For ψ_A_ = 0.5, stoichiometry allows for α = 1 for both species, so that extremely long AB repeating BCPs can be formed. At ψ_A_ = 2/3, the stoichiometry would allow for the formation of all ABA triblocks as there are two A chains present for each B chain. At ψ_A_ = 1/3, the inverse is true, allowing the formation of all BAB triblocks. Finally, at ψ_A_ = 0 and ψ_A_ = 1, the system is composed entirely of a B or A homopolymer, respectively. We can then approximate various parts of the ODT envelope using mixtures of these different components. In Figure 7, we use RPA to plot the disordered phase spinodal boundary (above or coincident with the ODT) for the fully reactive telechelic blend as well as for binary blends of homopolymers, triblocks, and the AB repeating polymer.

**Figure 8:**
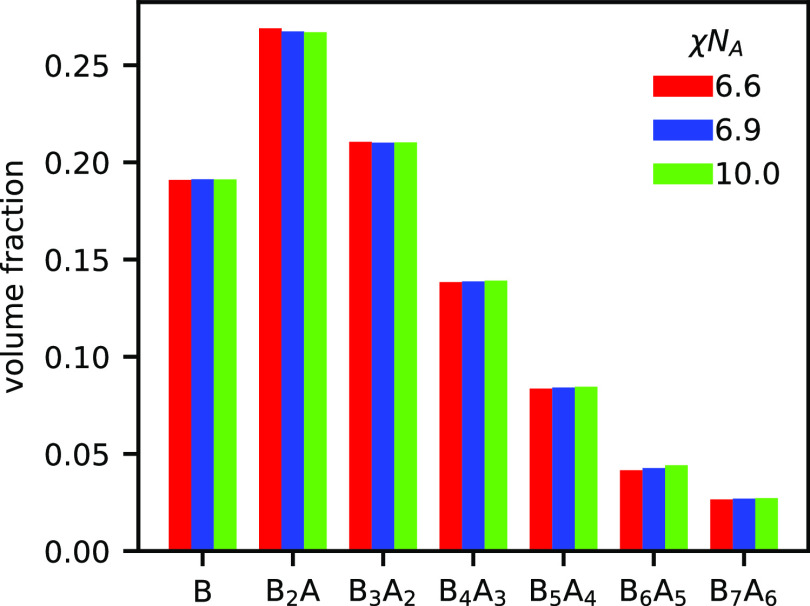
Distribution of products found in the telechelic blend at ψ_A_ = 0.32, *h*/χ*N*_A_ = 2, and various χ*N*_A_. For χ*N*_A_ = 6.6, the blend forms a homogeneous disordered phase. For χ*N*_A_ = 6.9 or 10.0, the blend self-assembles into HEX.

The unreactive binary blends match reasonably closely to the fully reactive telechelic system. This is somewhat surprising because the telechelic system is composed of a wide array of different BCP products. In Figure 8, we plot the full distribution of products at ψ_A_ = 0.32 and χ*N*_A_ = 6.6, 6.9, or 10.0. At χ*N*_A_ = 6.6, the system is still in the disordered phase, whereas at the two higher χ*N*_A_ values, the HEX phase is stable. In all cases, triblock polymers make up less than 30% of the volume, despite the stoichiometry allowing for the near-complete formation of triblocks. Homopolymers remain a significant contribution at ≈19% of the volume, and higher-order BCPs that are B-terminated make up the remainder. Although the crude model of a triblock mixed with a homopolymer matches the ODT for the telechelic blend closely, it does not represent the actual distribution of products.

We are also able to evaluate the distribution of products at ψ_A_ = 0.5 and χ*N*_A_ = 7, which is just below the ODT. Considering products with a length of up to 50 telechelics accounts for only 25% of the mass in the system, which reveals that the system is dominated by very long BCPs.

**Figure 9:**
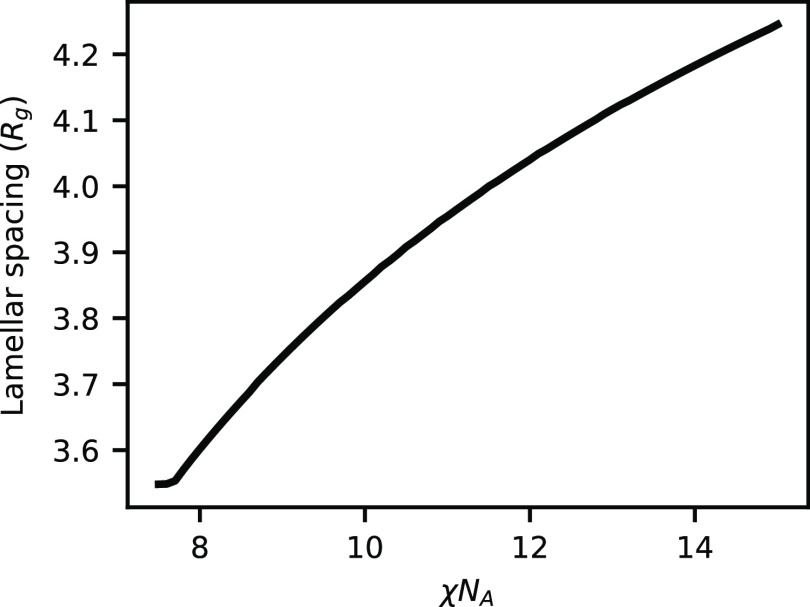
Domain spacing of the lamellar phase as a function of χ*N*_A_ at *h*/χ*N*_A_ = 2.0 and ψ_A_ = 0.5.

As a final comparison to the weak-bonding case, we plot the domain spacing of the lamellar phase at ψ_A_ = 0.5, as shown in Figure 9. For high bond strength, the domain spacing increases with increasing χ*N*_A_, which is the opposite trend that occurs at weak bonding. Additionally, the magnitude of change in domain spacing is much smaller at strong bonding compared to weak bonding. We can attribute the difference between the two regimes to the mechanisms that cause the domain spacing to change. At weak bonding, the domain size was largely affected by the conversion in the system and the amount of unreacted homopolymer that was present to swell the system. At strong bonding, the conversion is nearly unity in the lamellar phase, as shown in Figure 6. This means there is little to no homopolymer present to swell the system and the previous mechanism is no longer relevant. Instead, the brush physics at the lamellar interface dominate. In this picture, as χ*N*_A_ increases, the chains stretch away from the interface to reduce the interfacial area per unit volume, leading to increased domain spacing.^51^

### Intermediate Bonding

**Figure 10:**
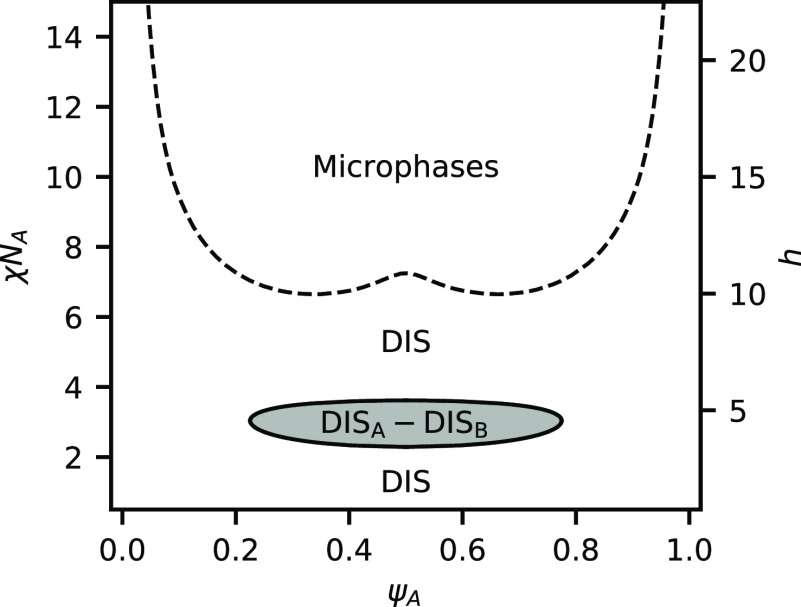
Phase diagram for a binary blend of heterobonding telechelic homopolymers at *h*/χ*N*_A_ = 1.5. Shaded regions indicate two-phase coexistence, while white regions indicate a single-phase is present. Only the stability limit of the homogeneous DIS phase to microphases is shown by the RPA analysis.

Thus far, we have considered supramolecular bonds that are relatively strong or weak compared to segregation strength. We now consider the transition between the two regimes and examine an intermediate bond strength of *h*/χ*N*_A_ = 1.5, for which the phase diagram is illustrated in Figure 10. The phase diagram shows features from both the weak bonding and strong bonding phase portraits. For 2 < χ*N*_A_ < 3.6, it is possible to phase separate into two DIS phases, similar to the weak-bonding case. This region has both UCST and LCST character and closes for χ*N*_A_ > 3.6, where a single DIS becomes stable. As χ*N*_A_ is further increased, the system undergoes another transition, but this time into microphases. We do not perform a full numerical SCFT investigation of this region, but the RPA reveals that the disordered phase has instabilities at nonzero wave vector *k*, indicating the formation of microphases. Furthermore, the shape of the phase boundary is highly reminiscent of that from *h*/χ*N*_A_ = 2 and we expect very similar stability windows. Such rich and reentrant phase behavior in the intermediate bonding strength regime was previously identified by RPA analysis of the binary telechelic system.^52^

**Figure 11:**
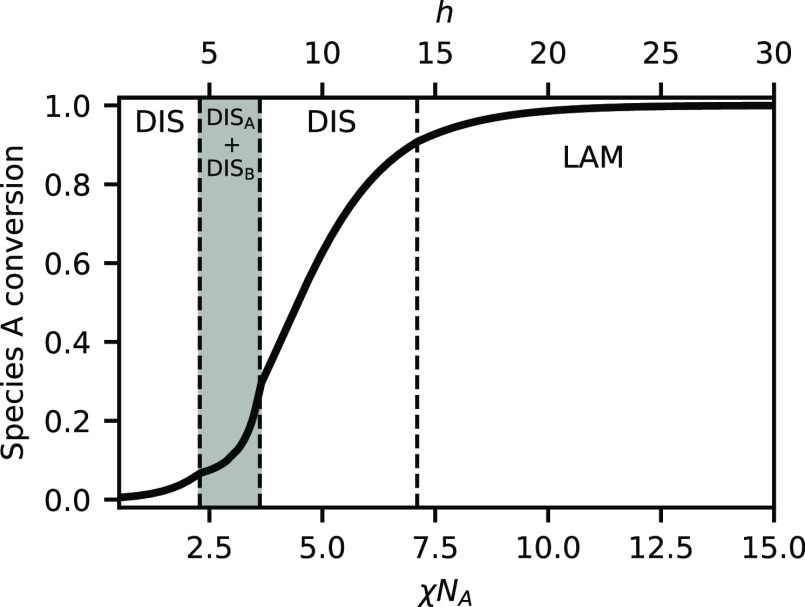
Species A conversion, α_A_, versus χ*N*_A_ (solid dark line) at *h*/χ*N*_A_ = 1.5 and ψ_A_ = 0.5. Shading and text labels indicate the stable phase(s). Vertical dashed lines indicate phase boundaries.

To understand this complex phase portrait, we again turn to the reaction equilibrium. Figure 11 shows the conversion of species A at ψ_A_ = 0.5 for varying χ*N*_A_. As in previous cases, conversion increases with increasing χ*N*_A_. At χ*N*_A_ ≈ 2.25, the conversion of species A is α_A_ ≈ 0.07, corresponding to an approximately 10% volume fraction of copolymer. This is an insufficient quantity of copolymer to prevent phase separation, but it is enough to delay phase separation from the unreactive critical point of χ*N*_A_ = 2. As χ*N*_A_ is further increased above 2.25, the conversion continues to increase, in contrast to the weak-bonding case, where conversion started to decrease upon phase separation. In this intermediate bonding case, the phase segregation near the critical point is weak and the A-rich phase is composed of at least 20% component B. The equilibrium constant is also sufficiently large so that forming BCP products is still favored, and conversion continues to increase. A careful examination of the conversion reveals that the phase separation does slightly depress conversion compared to a hypothetical scenario of a well-mixed single phase, but this effect is rather weak.

As χ*N*_A_ is increased through the two-phase window, eventually enough copolymer is formed to recompatibilize the two phases at χ*N*_A_ ≈ 3.6. The conversion at this point is α_A_ ≈ 0.27, which corresponds to a combined homopolymer volume fraction of 52%. This is consistent with previous theoretical studies of A homopolymer + B homopolymer + AB diblock that found that approximately 45% volume fraction of diblock copolymer was sufficient to compatibilize homopolymers^53^ when the homopolymer had half the length of the diblock, as in this study. For χ*N*_A_ > 3.6, the conversion plot strongly resembles that of the strong bonding case, and eventually the blend self-assembles into a lamellar phase. The trends in lamellar domain spacing also mimic those of the strong bonding case, and domain spacing increases monotonically with increasing χ*N*_A_.

The phase behavior at this intermediate value of *h*/χ*N*_A_ is indicative of being near a Lifshitz tricritical point, where microphase separation, macrophase separation, and a single disordered phase meet at a single point. It is known from unreactive polymer blends that SCFT fails dramatically near the Lifshitz point and fluctuations stabilize bicontinuous microemulsions.^54−56^ We expect such fluctuations to also be present in this system, but we do not speculate further on their effects. In principle, it is possible to include fluctuation effects in our model via field-theoretic simulations, which would also include cyclic copolymer species, but we defer such efforts to future work.

### Unequal Telechelic Polymer Lengths

**Figure 12:**
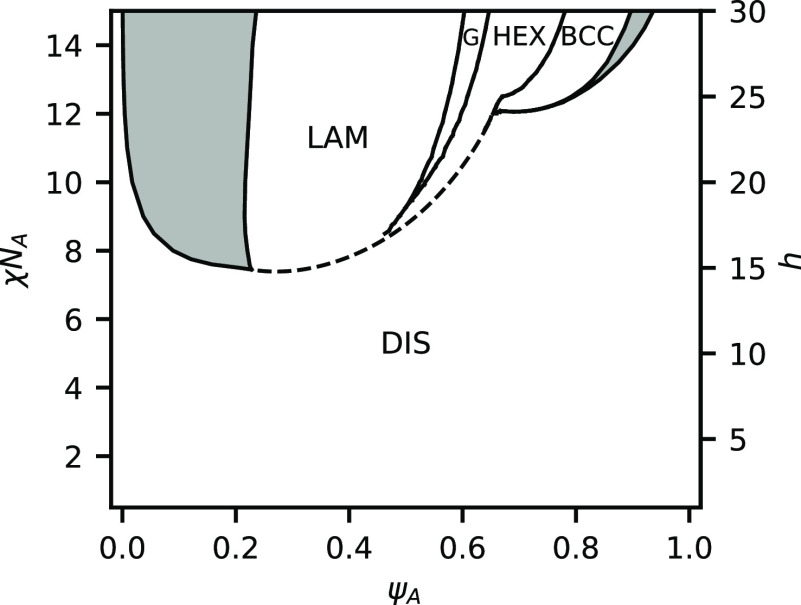
Phase diagram for a binary blend of heterobonding telechelic homopolymers at *h*/χ*N*_A_ = 2 with *N*_B_/*N*_A_ = 0.5. Shaded regions indicate two-phase coexistence, while white regions indicate a single-phase is present. Dashed line indicates the RPA spinodal boundary.

Up to this point, we have only considered blends where the two telechelic homopolymers are of equal length. We next consider the case where the B telechelic is half as long as the A homopolymer, *N*_B_/*N*_A_ = 0.5. Upon breaking the molar mass symmetry of the two polymers,the compositional symmetry of the phase diagram is correspondingly broken. Figure 12 shows the phase diagram for *N*_B_/*N*_A_ = 0.5 and *h*/χ*N*_A_ = 2. The phase diagram is dominated by the lamellar phase, which is stable as a pure phase or in coexistence with a disordered phase for ψ_A_ < 0.5. This is to be expected, as the analogous unreactive system consists of a BAB triblock that has an equal number of total A and B segments, blended with a B homopolymer. A similar system of symmetric AB diblock blended with B homopolymer also produced a region of LAM stability as well as LAM and DIS coexistence.^46,57^ For ψ_A_ > 0.5, other phases form, including GYR, HEX, and BCC. We do not include coexistence regions between microphases but rather draw solid lines where the free energies of the two phases cross. Coexistence regions between microphases have widths less than 0.01.

**Figure 13:**
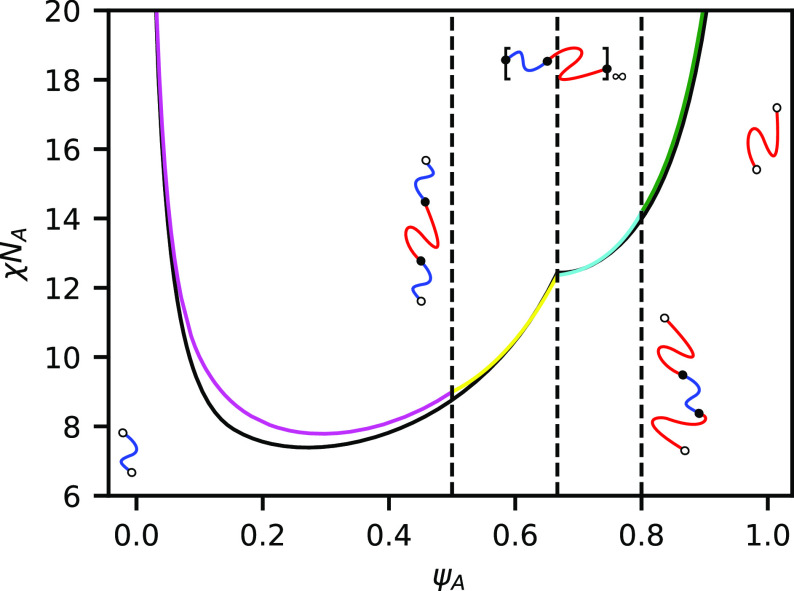
Order–disorder spinodal boundaries computed via RPA for the telechelic blend system with *N*_B_/*N*_A_ = 0.5 (black) and multiple unreactive binary blends, including: ABA triblock and A homopolymer (green), ABA triblock with infinitely repeating (AB) multiblock (cyan), BAB triblock with infinitely repeating (AB) multiblock (yellow), and BAB triblock with B homopolymer (magenta).

The asymmetric two-lobe structure of the phase diagram can also be understood by a comparison with unreactive analogous. The cusp occurs at ϕ_A_ = 2/3, which is the stoichiometric composition to form repeating AB BCPs. Figure 13 compares the telechelic blend spinodal boundary to that of various unreactive blends similar to that in Figure 7. Note, however, that the actual distribution of products is likely significantly different than the unreactive analogous considered here. This point was demonstrated earlier for equal length telechelic homopolymers.

## Comparison with Experiments

One of the earliest experimental investigations of the phase behavior of telechelic blends is due to Russell and co-workers.^9^ They used small-angle X-ray scattering to study blends of telechelic polyisoprene and polystyrene that were end-functionalized with amino and acid groups, respectively. The acid groups considered were carboxylic and sulfonic acids. Supramolecular bonds can form when an acid protonates an amino group, which induces an ionic bond. Bulk polyisoprene and polystyrene have dielectric constants near 3, so it is very unfavorable for unpaired ions to exist. For the bonds to dissociate, the reverse proton transfer must occur so that the neutral amine and acid can separate from one another. Evidence for the partial conversion of acid and base to paired ions was found by FT-IR in later work by Iwasaki and co-workers.^22^

The scattering data from amino-terminated polyisoprene blended with carboxylic acid-terminated polystyrene are consistent with the weak-bonding picture presented in this work. The authors observed microphase formation at low temperatures (high χ) and that upon heating, the blend would undergo spinodal decomposition and macroscopically phase separation, analogous to decreasing χ*N*_A_ in Figure 1. Additionally, the domain size of the microphase was found to significantly increase with increasing temperature (decreasing χ*N*_A_), consistent with weak-bonding domain size trends, as shown in Figure 3. The authors showed that the change in domain size was too large to be attributed to thermal expansion; therefore, the change in domain size due to homopolymer swelling demonstrated in this work may play a role.

When the carboxylic acid groups were replaced with sulfonic acid groups, Russell and co-workers found qualitatively different behavior. The blend again formed a microphase at low temperature, but upon heating, it became disordered rather than undergoing spinodal decomposition. This is consistent with the strong-bonding phase behavior presented in Figure 5. Additionally, the domain spacing of the microphase had a much weaker dependence on temperature compared with the blend with carboxylic acid. The domain size slightly decreased with increasing temperature, consistent with the trends in domain size at strong bonding in Figure 9. The blend with sulfonic-acid-functionalized polystyrene thus behaves like a polymer in the strong bonding regime predicted in this work. Furthermore, sulfonic acid is a stronger acid than carboxylic acid; therefore, the effective equilibrium constant should be larger for the sulfonic acid-amine pairing. Finally, Russell and coauthors showed that if the length of the polystyrene polymers was increased, then the system would again macrophase separate at high temperature, consistent with the intermediate-bonding phase diagram in Figure 10. This is consistent with previous theoretical investigations that showed that increased polymer length dilutes the concentration of end groups and leads to an effectively weaker equilibrium constant.^26^

There have been multiple subsequent reports that utilize the same acid-amine chemistry as Russell and co-workers, but swap the isoprene monomer with a different chemical species, including ethylene oxide, isobutylene, and dimethylsiloxane.^22,58,59^ In addition to SAXS, these authors have performed FT-IR, proton NMR, TEM, and rheological measurements on their samples to provide further evidence for BCP formation from the starting homopolymers and the presence of an ODT. One group of authors was also able to estimate the approximate values of χ*N*_A_ for which the ODT occurs, which matches closely to the value we predict at equal composition, as shown in Figure 5.^58^ In all of the referenced papers except those by Russell and co-workers, only equimolar blends of telechelic polymers were considered, so much of the phase space remains unexplored.

In addition to the acid base supramolecular interactions, there have been investigations of telechelic polymers that interact through hydrogen bonding.^60,61^ These investigations are not as extensive as the previously discussed work but were able to show evidence of microphase formation via SAXS and TEM. Unfortunately, there is not enough temperature-dependent data to compare these works with the bonding strength cases considered here.

## Conclusions

We have demonstrated that a wide variety of phase behaviors can be achieved with binary blends of heterobonding telechelic homopolymers. By properly tuning the relative strength of the bonding equilibrium constant to the segregation strength, it is possible to make the system behave like an unreactive homopolymer blend or a BCP melt. Although some of the microphase stability windows have unconventional shapes, these stability windows can be rationalized by considering the stoichiometry of the system. Additionally, the microphases that are formed from the telechelic blend can have highly variable domain sizes and differing dependence on temperature, depending on the relative strength of bonding and phase separation. The models presented here are able to quantitatively predict all of these phenomena and are consistent with previous experiments. This work confirms that CS-SCFT is a powerful theoretical and computational framework that can further guide the experimental investigation of supramolecular blends.
